# Incidence of intra-axial brain metastases in Eastern Finland

**DOI:** 10.1007/s11060-026-05842-7

**Published:** 2026-10-20

**Authors:** Samir Moniruzzaman, Susanna Rantala, Juhana Hakumäki, Tuomas Rauramaa

**Affiliations:** 1 Department of Neurosurgery, Kuopio University Hospital and Neurosurgery, Institute of Clinical Medicine, University of Eastern Finland, Kuopio, Finland; 2 Institute of Clinical Medicine, University of Eastern Finland, Kuopio, Finland; 3 Department of Clinical Radiology, Diagnostic Imaging Center, Kuopio University Hospital, Kuopio, Finland; 4 Department of Clinical Pathology, Kuopio University Hospital and Clinical Pathology and Forensic Medicine, Institute of Clinical Medicine, University of Eastern Finland, P.O. Box 1627, Kuopio, 70211 Finland

**Keywords:** Brain metastasis, Brain imaging, Intracranial metastatic disease, Incidence

## Abstract

**Purpose:**

Brain metastases (BM) incidence and characteristics remain poorly defined due to registry data being often incomplete, recording only primary tumors or unspecified metastases. The objective of this study was to examine BM incidence rates and characteristics confirmed by MRI in North Savo, Finland.

**Methods:**

In this retrospective study, North Savo residents with first MRI diagnosis of BM between 1 January 2004 and 31 December 2023 were identified through the radiology information system, irrespective of primary cancer diagnosis date. Medical and histopathological records were reviewed. Population person-years were estimated by summing annual end-year counts of all North Savo residents obtained from Statistics Finland. Crude incidence rates and age-standardized incidence rates (ASIRs), standardized to the 2013 European Standard population, were calculated. Temporal trends were assessed using age-adjusted Poisson regression.

**Results:**

Among 516 patients, 474 had histopathological verification. Cancers originating from lung and breast tissue accounted for 56.8% of BM cases. Overall crude incidence was 10.22 (95% CI 9.36–11.15) and ASIR was 9.20 (95% CI 8.42–10.04) per 100,000 person years. ASIR increased from 5.8 in 2004–2008 to 11.8 in 2019–2023. Age-adjusted Poisson regression estimated a 24% increase in incidence per five-year period (incidence rate ratio 1.24; 95% CI 1.14–1.34; *P* < 0.001). Age-specific incidence peaked at 33.1 per 100,000 person years among patients aged 70–74 years.

**Conclusions:**

MRI-confirmed BM incidence increased over 20 years, despite age standardization. Incidence was highest among older adults. These findings provide contemporary population-based epidemiological data of BM incidence.

## Introduction

Brain metastases (BM) are the most common tumors of the central nervous system (CNS), with nearly all malignancies capable of metastasizing to the brain [1, 2]. The most common primary tumors are cancers of lung, breast, melanoma, kidney, and colorectal origin [3, 4]. The lifetime incidence of BM in cancer patients is approximately 10–20% [2, 4]. Population studies, clinical series, surgical series, and autopsy studies have been used to characterize BM demographics [2]. Previous studies provide incidence estimates of BM around 8.3–24 per 100,000 persons per year [3, 5–8]. Considering that populations are aging globally while cancer therapies are continuously improving, more patients are surviving their primary disease. Consequently, the incidence of BM is expected to increase [2, 4]. BM are managed through a multimodal approach, including surgery, radiation therapy, chemotherapy, immunotherapy, and targeted therapies [9]. In clinical practice, computed tomography (CT) remains a valuable diagnostic modality for brain tumor imaging, particularly for the screening of patients with acute neurological deficits. Magnetic resonance imaging (MRI) is the primary imaging modality used to evaluate most intracranial lesions. Challenges in detecting and diagnosing BM include distinguishing them from mimics (e.g., primary tumors, infections), detecting small lesions, and differentiating treatment response from recurrence or progression [10]. Biopsy is often performed for histological confirmation, even in patients with prior history of malignant disease [11]. However, the primary tumor responsible for BM may remain unknown throughout treatment, follow-up, and even autopsy examination [12]. Microscopic examination of the BM typically reveals morphology reflecting the primary site, which distinguishes them from primary brain tumors.

Reliable classification of BM requires evidence of a known or suspected primary cancer, imaging findings (e.g., CT & MRI) consistent with BM, and histopathological confirmation of either the primary tumor or metastatic lesion. Because cancer registries do not capture BM, the population-level burden of intracranial metastatic disease is difficult to quantify. Registry data on BM are often variable in quality, with radiological, histological, and surgical data stored in separate databases. Finland’s well-organized healthcare system and high-quality healthcare registries provide comprehensive long-term follow-up data, with virtually no patients lost in follow-up. The objective of this study was to integrate data from these multiple registries and thereby assess the incidence and characteristics of MRI- and histopathologically confirmed BM over a 20-year period in the North Savo region.

## Materials and methods

### Data collection and selection

**Fig. 1:**
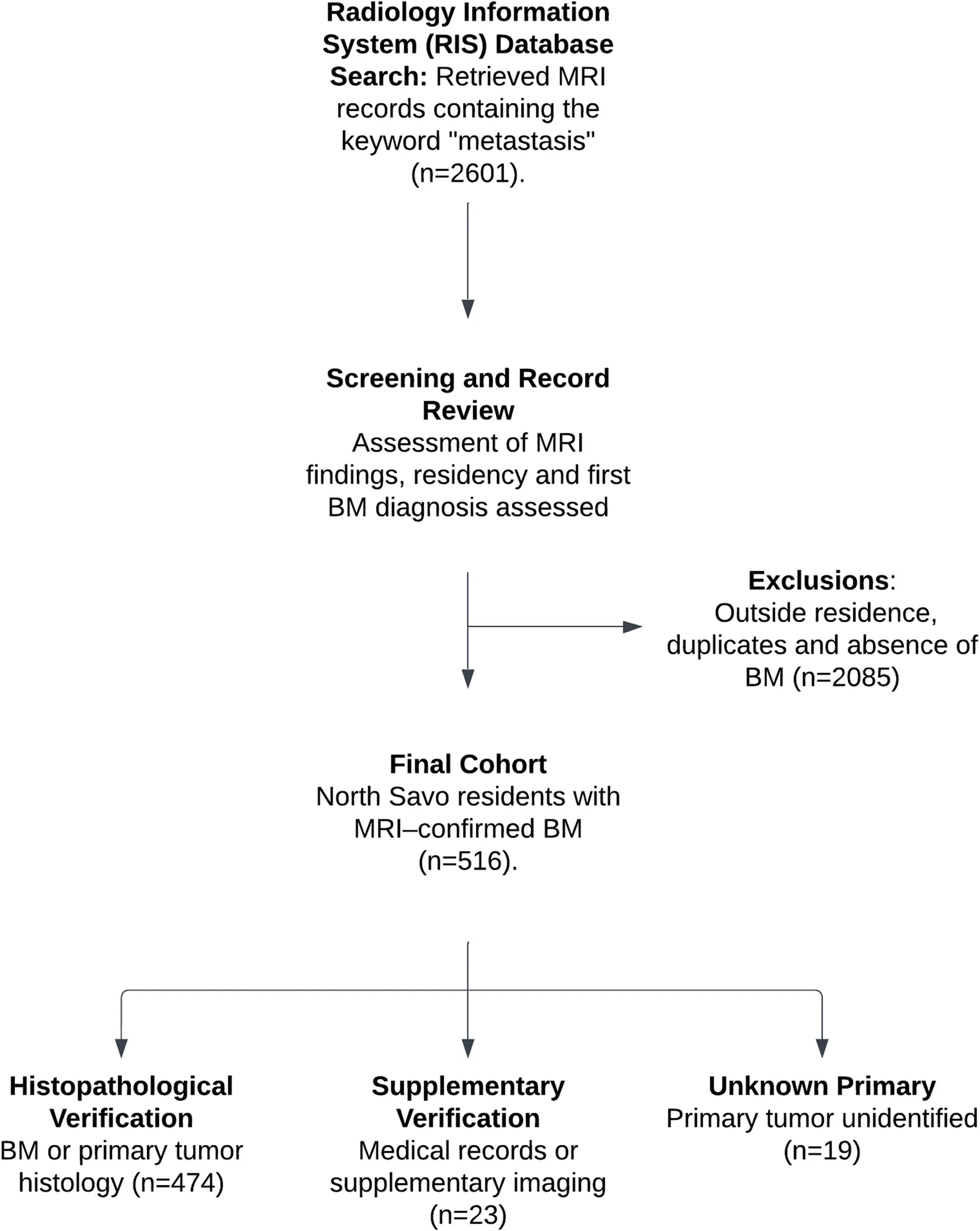
Patient selection flowchart. Histopathology of BM or primary tumor verified in 474 patients. Additional 23 patients verified through medical records or supplementary imaging. Primary tumor remained unidentified in 19 patients, including nine with inconclusive histopathological samples

Kuopio University Hospital (KUH) provides university hospital-level healthcare services to its catchment area. All regional MRI reports are logged into the local radiology information system (RIS) database. For this population based retrospective observational study, we focused on North Savo county, to ensure complete patient records. To identify patients with BM, we first conducted a search in the database for MRI reports with the various Finnish language linguistic derivatives of “metastasis” used in radiology reports (see Fig. 1). Our search provided a total of 2601 reports between 1 January 2004 to 31 December 2023, from which reports indicating an absence of BM (e.g., “no metastasis”) or residence outside North Savo were excluded. Patients were included at their first MRI with diagnosis of BM during the study period, irrespective of when primary cancer was diagnosed. North Savo residency was determined at BM diagnosis. Duplicate reports and subsequent MRI examinations were excluded. The confirmation of diagnosis, primary tumor site, and histological subtype was then verified by cross-referencing the MRI-cases with histopathological records, resulting in MRI-positive metastatic lesions which were linked to patient records. BM histopathology was preferred, but primary tumor histology was used when BM histology was unavailable. Cases with CNS tumors and non-solid tumors (e.g., primary glioblastoma, meningioma, hemangioblastoma, medulloblastoma, CNS lymphoma), and nonmalignant diseases (e.g., inflammation, Langerhans cell histiocytosis) were excluded. Patients with multiple BM were counted as one case according to the histopathological analysis of the primary tumor or BM. The data collection process is illustrated in Fig. 1.

### MR imaging review

All MR examinations were interpreted by an expert or fellow neuroradiologist using Picture Archiving and Communications System (PACS) workstations during patient management. All MRI reports were generated during clinical care and stored in the Radiology Information System (RIS) database. The typical brain tumor MRI protocol during the study period included axial T2-weighted imaging, coronal fluid-attenuated inversion recovery (FLAIR) imaging, axial diffusion-weighted imaging (DWI), and a 3D T1-weighted multi-planar reconstruction (MPR) sequence both before and after intravenous gadolinium contrast administration.

### Statistics Finland

Statistics Finland is Finland’s national statistical institute that produces impartial statistics on Finnish society. Population based data on the county of North-Savo was obtained through Finland’s national Population Information System through Statistics Finland, which cover registered permanent residents with a personal identity code and a municipality of residence in Finland. Over the study period the population of North Savo decreased from 256,740 (2004) to 248,190 (2023).

### Tumor characteristics

Tumor characteristics were obtained through histopathologic analysis linked to patient records, according to the World Health Organization (WHO) classification of tumors (5th edition). Lung cancer was classified according to histopathological analysis into adenocarcinoma, squamous cell, small cell, non-small cell not otherwise specified (NOS), and indefinite (unknown). Breast cancer was classified into four groups according to HER2 and hormone (estrogen and progesterone) receptor (HR) status: triple-negative (HR- and HER2-), HR- and HER2+, HR+ (either estrogen or progesterone) and HER2-, and lastly HR + and HER2+. Breast cancer reports lacking complete HER2 and HR classification were classified as unknown.

### Statistical analysis

Descriptive statistics and incidence calculations were performed using Microsoft Excel (Version 2606). Poisson regression and Fay-Feuer analyses were performed using IBM SPSS Statistics (Version 31.0). Continuous variables were summarized using median and interquartile range (IQR). Categorical variables were summarized as frequencies and percentages. BM cases were classified by age and calendar period at first MRI diagnosis of BM. Denominators comprised person-years for the general population of North Savo, irrespective of cancer status. Total and age-specific population person-years were estimated by summing annual year-end population counts for North Savo obtained from Statistics Finland during 2004–2023. Crude incidence rates and age-specific incidence rates were calculated by dividing BM case counts by the corresponding population person-years. Age-standardized incidence rates (ASIRs) were calculated by direct standardization to the 2013 European Standard Population (ESP2013). All age groups, including groups with no BM cases, were retained in age standardization. All rates were expressed per 100,000 person-years. Exact Poisson 95% confidence intervals (CIs) were calculated for crude and age-specific incidence rates and the Fay–Feuer gamma method was used for ASIRs [13].

Temporal trends were assessed by calculating ASIRs for consecutive five-year periods and fitting age-adjusted Poisson regression models of BM case counts with a log link. Age was included as a categorical variable using five-year age groups, with a final group aged ≥ 90 years. The log of North Savo population person-years in the corresponding age groups and periods was used as an offset. Calendar period was analyzed both as a linear trend to estimate the incidence rate ratio (IRR) per five-year period, and as a categorical variable with 2004–2008 as the reference. For comparison, a linear trend model without age adjustment was fitted using total BM counts for each period, with the log of corresponding total population person-years as an offset. IRRs are reported with 95% Wald CIs and two-sided Wald P values. The two age-adjusted models were compared using a likelihood-ratio test. A sensitivity analysis repeated the age-adjusted linear trend model after excluding 2004–2008. Age groups with no cases across all included periods were removed from age-adjusted regression, while zero-count observations in the remaining groups were retained.

## Results

### Study population

A total of 516 patients (47.9% male, 52.1% female) with MRI-detected BM from the North Savo region were identified between January 2004 and December 2023. The median age at diagnosis was 66 years (IQR 58–73; range 22–94 years). Among these 516 patients, cancer arising from lung tissue accounted for the largest proportion of all BM cases (32.0%), followed by breast cancer (24.8%), genitourinary (13.6%), gastrointestinal (10.1%), and melanoma (9.5%). Cancers arising from lung and breast tissue together accounted for 56.8% of cases. Patients with breast cancer developed BM at a younger median age than patients with lung, gastrointestinal, genitourinary, and melanoma primaries. Patients with an unknown primary were the oldest at diagnosis (median age 81 years). Detailed patient and tumor characteristics are presented in Table 1. Histopathological verification was obtained in 474 cases, while 23 additional cases were verified through prior medical records or supplementary radiological imaging (e.g., CT scans) (Fig. 1). The remaining 19 patients had MRI positive BM of unknown primary origin, with no prior medical history of malignancy or definitive histological verification. Histopathological samples were obtained in nine of these patients but remained inconclusive.

**Table 1:** Patient and tumor characteristics of North Savo BM cohort, 1 January 2004–31 December 2023

Patient characteristics	*n* (%)	
Total patients	516 (100)	
Male sex	247 (47.9)	
Female sex	269 (52.1)	
Age at diagnosis, median (IQR)	66 (58–73)	
Age at diagnosis, range	22–94	
**Primary tumor**	**n (%)**	**Median age (IQR)**,** years**
Lung	165 (32.0)	66 (61–71)
Adenocarcinoma	88 (17.1)	64.5 (60–69)
Small cell	31 (6.0)	66 (62–70)
Squamous cell	23 (4.5)	67 (63–73)
Non-small cell NOS	8 (1.6)	NR
Lung unknown	15 (2.9)	70 (63–80)
Breast	128 (24.8)	61 (52–70)
HR+/HER2-	60 (11.6)	61 (54–70)
HR+/HER2+	28 (5.4)	58 (49–75.25)
HR-/HER2+	18 (3.5)	60.5 (51.75–70)
Triple negative	16 (3.1)	59.5 (46.25–66.25)
Breast unknown	6 (1.2)	NR
Genitourinary	70 (13.6)	68 (61.75–74)
Kidney	29 (5.6)	67 (60.5–75)
Prostate extra-axial	26 (5.0)	69 (64–72.25)
Prostate intra-axial	6 (1.2)	NR
Bladder	8 (1.6)	NR
Testicular	1 (0.2)	NR
Gastrointestinal	52 (10.1)	68 (58–76)
Colorectal	31 (6.0)	68 (58–77)
Esophagus	9 (1.7)	NR
Gastric	5 (1.0)	NR
Hepatobiliary	4 (0.8)	NR
Pancreatic	3 (0.6)	NR
Melanoma	49 (9.5)	69 (59.5–76)
Gynecologic	18 (3.5)	71 (57–74)
Adnexal	14 (2.7)	NR
Uterus	4 (0.8)	NR
Oral cavity	8 (1.6)	NR
Sarcoma	4 (0.8)	NR
Thyroid	2 (0.4)	NR
Nasal	1 (0.2)	NR
Unknown primary	19 (3.7)	81 (68–88)

Data presented as n (%) and median age (IQR). Percentages are based on all 516 patients with BM. Age refers to first MRI diagnosis of BM. IQR reported only for groups with ≥ 15 cases. (NR, not reported, due to small sample size). Abbreviations: BM, brain metastases; IQR = interquartile range; HER2, human epidermal growth factor receptor 2; HR, hormone receptor. Extra-axial = dura and skull, intra-axial = within brain parenchyma

### Incidence by primary tumor

Over the study period, the crude incidence of MRI-detected BM among all North Savo residents was 10.22 per 100,000 person-years (95% CI 9.36–11.15), and the ASIR was 9.20 per 100,000 person-years (95% CI 8.42–10.04). Annual crude incidence in this population ranged from 3.9 per 100,000 person-years (95% CI 1.9–7.2) in 2004 to 17.3 per 100,000 person years (95% CI 12.5–23.3) in 2023.

Incidence rates by primary tumor site were calculated using population person-years among all North Savo residents. Primary tumor group percentages were based on all 516 patients with BM. Cancer arising from lung tissue was the most common primary tumor associated with BM, accounting for 32.0% (165/516) of all BM cases. BM originating from cancer of lung tissue had the highest crude incidence in the general population of North Savo (3.27 per 100,000 person-years; 95% CI 2.79–3.81) and ASIR (2.89 per 100,000 person-years; 95% CI 2.47–3.38). Breast cancer represented the second largest primary tumor group, accounting for 24.8% (128/516) of all BM, with a crude incidence of 2.54 per 100,000 person-years (95% CI 2.12–3.02) and an ASIR of 2.35 per 100,000 person-years (95% CI 1.96–2.80). Together, cancers of lung and breast tissue accounted for more than half of all BM (56.8%, 293/516). Genitourinary (13.6%), gastrointestinal (10.1%), and melanoma (9.5%) were the next most frequent primary tumor groups but demonstrated substantially lower crude and ASIR than lung and breast cancers.

Among BM originating from cancer of lung tissue, adenocarcinoma was the predominant histological subtype, accounting for 53% (88/165) of cases, followed by small cell carcinoma (19%, 31/165) and squamous cell carcinoma (14%, 23/165). Among breast cancer-derived BM, HR+/HER2 − tumors represented the largest molecular subtype (47%, 60/128), followed by HR+/HER2 + tumors (22%, 28/128), whereas HR−/HER2 + and triple-negative disease accounted for 14% (18/128) and 13% (16/128) of cases, respectively **(**Table 2**)**.

**Table 2:** Crude and age-standardized incidence rates of BM by primary tumor group in North Savo (per 100,000 person-years), 1 January 2004–31 December 2023

Primary tumor	*n* (%)	Crude Rate	95% CI	ASIR	95% CI
All BM	516 (100.0)	10.22	9.36–11.15	9.20	8.42–10.04
Lung	165 (32.0)	3.27	2.79–3.81	2.89	2.47–3.38
Adenocarcinoma	88 (17.1)	1.74	1.40–2.15	1.55	1.24–1.92
Small cell	31 (6.0)	0.61	0.42–0.87	0.54	0.37–0.77
Squamous cell	23 (4.5)	0.46	0.29–0.68	0.40	0.26–0.62
Non-small cell NOS	8 (1.6)	0.16	0.07–0.31	NR	NR
Lung unknown	15 (2.9)	0.30	0.17–0.49	0.26	0.14–0.44
Breast	128 (24.8)	2.54	2.12–3.02	2.35	1.96–2.80
HR+/HER2-	60 (11.6)	1.19	0.91–1.53	1.08	0.82–1.40
HR+/HER2+	28 (5.4)	0.55	0.37–0.80	0.54	0.35–0.78
HR-/HER2+	18 (3.5)	0.36	0.21–0.56	0.33	0.19–0.53
Triple negative	16 (3.1)	0.32	0.18–0.51	0.30	0.17–0.50
Breast Unknown	6 (1.2)	0.12	0.04–0.26	NR	NR
Genitourinary	70 (13.6)	1.39	1.08–1.75	1.23	0.96–1.56
Kidney	29 (5.6)	0.57	0.38–0.83	0.50	0.34–0.73
Prostate extra-axial	26 (5.0)	0.52	0.34–0.75	0.46	0.30–0.69
Prostate intra-axial	6 (1.2)	0.12	0.04–0.26	NR	NR
Bladder	8 (1.6)	0.16	0.07–0.31	NR	NR
Testicular	1 (0.2)	0.02	0.00–0.11	NR	NR
Gastrointestinal	52 (10.1)	1.03	0.77–1.35	0.92	0.69–1.22
Colorectal	31 (6.0)	0.61	0.42–0.87	0.54	0.37–0.78
Esophagus	9 (1.7)	0.18	0.08–0.34	NR	NR
Gastric	5 (1.0)	0.10	0.03–0.23	NR	NR
Hepatobiliary	4 (0.8)	0.08	0.02–0.20	NR	NR
Pancreatic	3 (0.6)	0.06	0.01–0.17	NR	NR
Melanoma	49 (9.5)	0.97	0.72–1.28	0.89	0.66–1.18
Gynecologic	18 (3.5)	0.36	0.21–0.56	0.32	0.19–0.52
Adnexal	14 (2.7)	0.28	0.15–0.47	NR	NR
Uterus	4 (0.8)	0.08	0.02–0.20	NR	NR
Oral cavity	8 (1.6)	0.16	0.07–0.31	NR	NR
Sarcoma	4 (0.8)	0.08	0.02–0.20	NR	NR
Thyroid	2 (0.4)	0.04	0.00–0.14	NR	NR
Nasal	1 (0.2)	0.02	0.00–0.11	NR	NR
Unknown	19 (3.7)	0.38	0.23–0.59	0.34	0.20–0.54

Data are presented as n (%), and incidence rates per 100,000 person-years with 95% confidence intervals (CIs). Percentages are based on all 516 patients with BM. Crude incidence rates use total population person-years among North Savo residents of all ages and both sexes, irrespective of cancer status. Age-standardized incidence rates (ASIRs) use age-specific person-years from the same population and direct standardization to the 2013 European Standard Population (ESP2013). CIs were calculated using exact Poisson method for crude rates and the Fay-Feuer gamma method for ASIRs. ASIRs were not reported for groups with < 15 cases because of statistical instability (NR, not reported). Abbreviations: BM, brain metastases; HER2, human epidermal growth factor receptor 2; HR, hormone receptor. Extra-axial, dura and skull; intra-axial, brain parenchyma

### Age-specific incidence

**Fig. 2:**
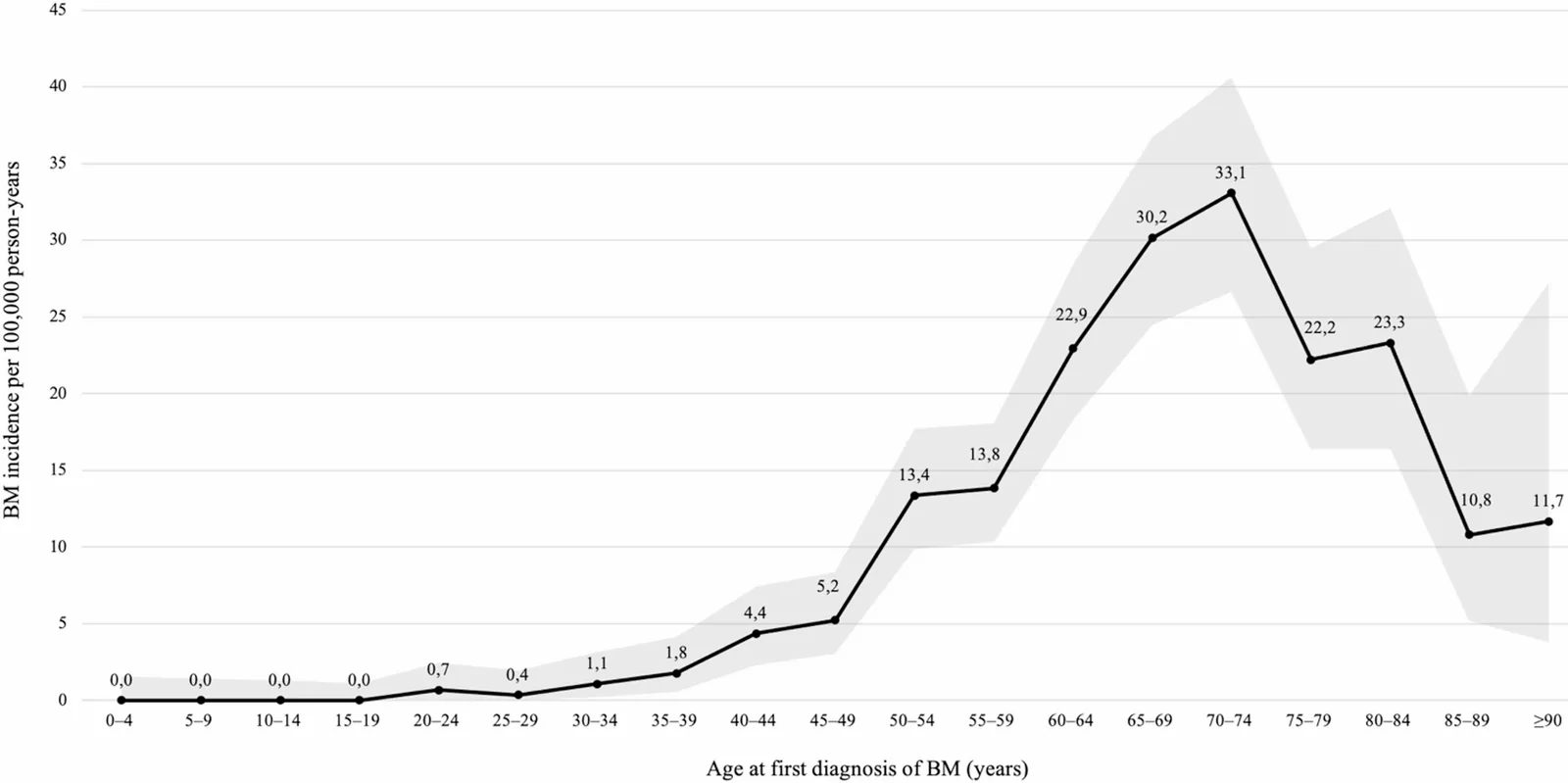
Age-specific incidence rates of brain metastases (BM) per 100,000 person-years in North Savo, 1 January 2004–31 December 2023. Points show rates and shaded area indicates exact Poisson 95% confidence intervals. Denominators were age-specific person-years among all North Savo residents. Age was recorded at first MRI diagnosis of BM

Age-specific BM incidence was calculated using person-years among North Savo residents in each corresponding age group. No BM were recorded among residents younger than 20 years, and incidence remained low until approximately 40 years of age. Thereafter, incidence increased from the fifth decade onward, rising from 13.4 per 100,000 person-years in the 50–54-year age group to a peak of 33.1 per 100,000 person-years in residents aged 70–74 years. Incidence subsequently declined but remained above 20 per 100,000 person years in the 75–79- and 80–84-year age groups before decreasing further among patients aged 85 years and older. Overall, the incidence increased approximately sixfold between the 45–49 and 70–74-year age groups (Fig. 2).

### Temporal trends in BM incidence

**Fig. 3:**
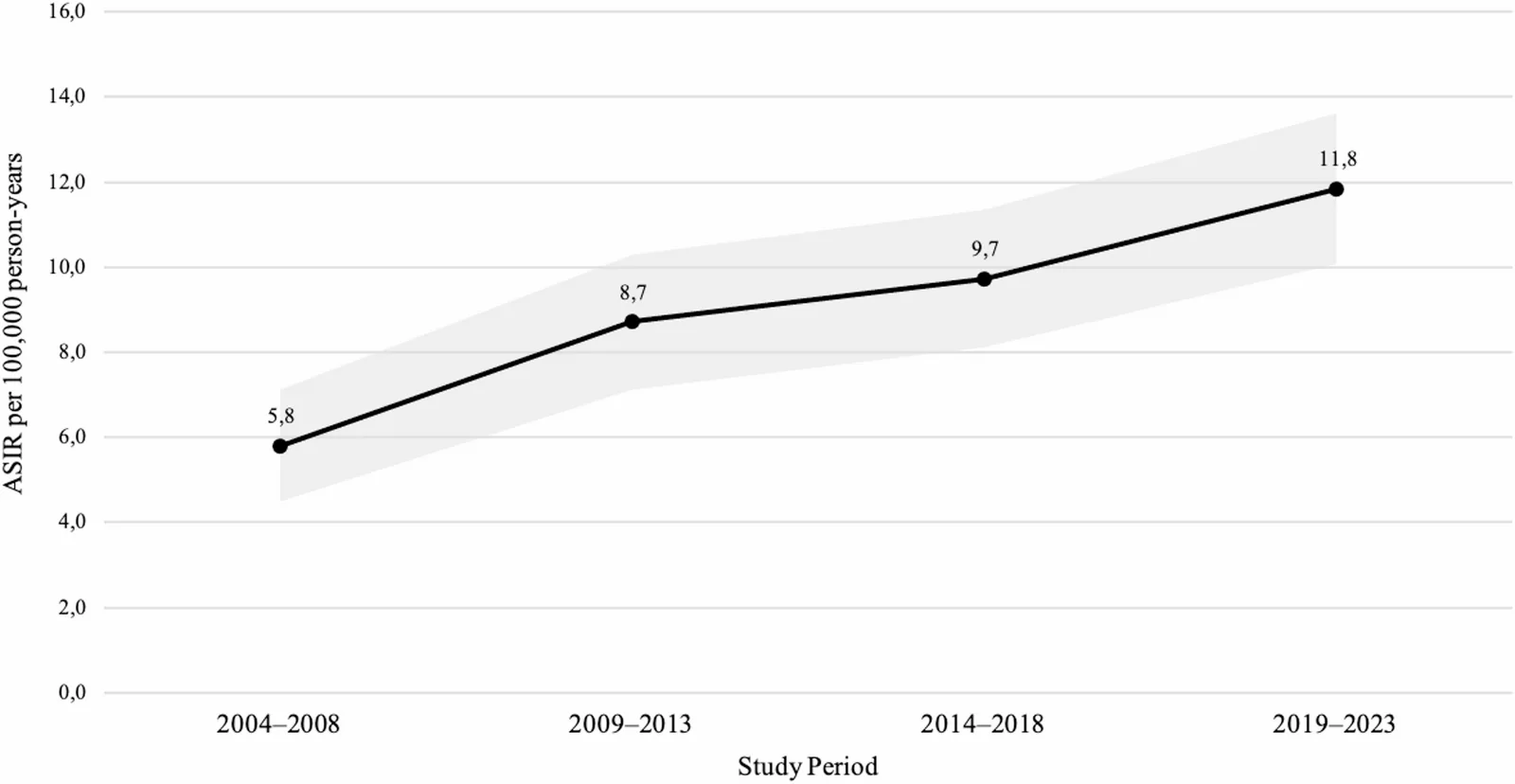
Temporal trends in age-standardized incidence (ASIRs) of of brain metastasis (BM) per 100,000 person years in North Savo, 1 January 2004–31 December 2023. ASIRs used age-specific person-years among all North Savo residents in each five-year period and were standardized to the 2013 European Standard Population. Points show ASIRs. Shading indicates 95% confidence intervals using Fay-Feuer gamma method. Cases were classified by age and date at first MRI diagnosis of BM

The overall ASIR of BM among North Savo residents increased over the study period from 5.8 per 100,000 person-years during 2004–2008 to 11.8 per 100,000 person-years during 2019–2023, approximately twice the initial rate (Fig. 3). The largest increase occurred between the first and second five-year periods, followed by a more gradual increase. In the age-adjusted model with calendar period as a categorical variable, IRRs compared with 2004–2008 were 1.46 (95% CI 1.09–1.94; *P* = 0.010) for 2009–2013, 1.64 (95% CI 1.24–2.17; *P* < 0.001) for 2014–2018 and 2.02 (95% CI 1.54–2.64; *P* < 0.001) for 2019–2023. The linear trend models estimated average increases per five-year period of 31% in crude BM incidence (IRR 1.31; 95% CI 1.21–1.42; *P* < 0.001) and 24% after age adjustment (IRR 1.24; 95% CI 1.14–1.34; *P* < 0.001). The age-adjusted linear trend remained significant after excluding 2004–2008 (IRR 1.17; 95% CI 1.04–1.32; *P* = 0.007). Among the age-adjusted models, changing calendar period to a categorical variable did not significantly improve fit compared with the linear trend model (likelihood-ratio χ²=1.36; df = 2; *P* = 0.51).

## Discussion

In this population-based study, we found the ASIR of BM increased steadily over the 20-year study period despite age standardization. Age-specific incidence increased with advancing age and peaked among patients aged 70–74 years. Our findings regarding the primary tumor site were in line with the prior literature, with the most common primary sites being the lung tissue, breast, melanoma, colorectal, and kidney [3, 4, 14]. Cancer originating from lung and breast tissue accounted for more than half of BM, with lung adenocarcinoma and breast HR+/HER2- representing the predominant histological and molecular subtypes. In addition, we determined the incidence of other primary cancer types that rarely cause BM.

In the present study, the overall crude incidence was 10.2 per 100,000 person years. This estimate is in-line with previously reported population-based incidence rates, which provide incidence estimates of BM around 8.3–24 per 100,000 persons per year [3, 5–8]. This comparable incidence rate supports the completeness of case ascertainment through the method used.

Population-based estimates of the incidence of BM remain limited. Previous studies have focused on selected primary cancer types [4], or relied on data from the Surveillance, Epidemiology, and End Results (SEER) program. A key limitation of SEER-based studies is that they capture only synchronous BM at initial cancer diagnosis, excluding metachronous BM and therefore possibly underestimating the overall population burden [14–16]. Using SEER data, previous population-based studies estimated the overall ASIR of synchronous BM to be approximately 7 per 100,000 persons between 2010 and 2015 and 7.1 per 100,000 persons between 2015 and 2019 [15, 16]. In comparison, the overall ASIR observed in our study was 9.2 per 100,000 persons. This slightly higher estimate is possibly explained by differences in case ascertainment. Unlike SEER-based studies, our analysis captured both synchronous and metachronous BM, providing a more comprehensive assessment of the population burden.

We observed an increase in the overall ASIR of BM over the 20-year study period, with approximately twice the initial rate between the first and last five-year periods. A similar increase was reported in a Swedish population-based study of hospitalized patients with BM between 1987 and 2006 [7]. In comparison, a recent SEER-based study reported a modest decline in the ASIR of synchronous BM between 2010 and 2019 (annual percent change − 0.6%) at primary cancer diagnosis [17].

Annual crude incidence showed a similar upward trend, from 3.9 per 100,000 person-years in 2004 to 17.3 in 2023. Age adjustment reduced the estimated average increase from 31% to 24% per successive five-year period, suggesting that population aging contributed partly to the crude trend. The increase remained statistically significant after age adjustment, suggesting that population aging alone is unlikely to explain the observed trend. The age-adjusted linear trend also remained significant after excluding the first period between 2004 and 2008, suggesting that the upward trend was not driven alone by the lower observed incidence in the first period. However, age adjustment may not fully account for differences in age within each age-group.

Increased access to MRI and lower thresholds for neuroimaging may have improved the detection of small and asymptomatic BM compared with CT [1, 4, 7, 10]. Advances in systemic cancer therapies have prolonged survival [18], potentially increasing the time at risk for developing BM [2, 4]. Greater clinical awareness of BM, improved referral pathways and increased registry completeness may also have contributed, particularly during the early years of this study. However, MRI availability and use were not assessed directly, and the interval between primary cancer and first MRI diagnosis of BM was not assessed, so their effects to the observed trend remain unclear. These factors may partly explain the observed increase of BM incidence.

We found that BM originated primarily from cancer of lung tissue, followed by breast cancer and melanoma, consistent with previous studies [2–4, 14]. Cancers originating from lung and breast tissue accounted for more than half of all BM. This is expected given both the high incidence of lung cancer and its marked propensity for intracranial dissemination [7, 14]. A recent SEER-based study reported a slightly higher ASIR of 4.6 per 100,000 for BM from NSCLC and 0.87 per 100,000 for SCLC, while the overall incidence of lung cancer BM decreased over time [19]. Modest improvements in survival from systemic therapies with poor intracranial penetration have prolonged lifespans, increasing the opportunity for BM to develop [20].

Prior studies have reported a higher risk of BM among patients with triple-negative and HR-/HER2 + breast cancer compared to HR+/HER2- disease [3, 21, 22]. In our cohort, HR+/HER2- was the most common subtype among patients with breast cancer originating BM. Our findings describe the subtype distribution among patients with BM and not estimate the risk of BM within each breast cancer subtype. The predominance of HR+/HER2- may reflect its higher prevalence among breast cancer diagnoses. A longer disease course may also allow more time for BM to develop, although this was not assessed in our study.

In our study, the incidence of BM increased markedly with age and reached its highest level among individuals aged 70–74 years. Previous studies have reported similar results, with the majority of patients diagnosed at 60–79 years old [15–17]. New therapeutics, advanced imaging, and improved screening have led to improved survival after primary cancer diagnosis [23]. Consequently, improved survival provides a longer time at risk of metastatic spread to the brain [2]. The decline in incidence of BM among patients over 75 years may reflect competing mortality due to other causes, in addition to the reduced use of neuroimaging in cases where it is deemed unlikely to alter prognosis or treatment decisions, including patients in palliative care. Furthermore, patient preferences may also have influenced decisions to pursue neuroimaging and inconclusive MRI findings may not be necessarily followed by biopsy and histopathological analysis to distinguish BM from other radiological mimics.

This study has several strengths, including its population-based design, combined review of clinical, radiological, and histopathological records, and a 20-year study period with virtually complete follow-up. General population counts from Statistics Finland provided accurate denominators for regional incidence estimates. This study also has limitations. Its retrospective design is prone to missing data, and findings from a single Finnish region may not be fully generalizable. Histopathological verification of BM or the primary tumor was available for 474 of 516 patients, while the primary tumor remained unidentified in 19. BM diagnosed without MRI were not included, and the completeness of case ascertainment could not be assessed. Population-person years were estimated from annual year-end counts, and age adjustment may not fully account for age differences within each group.

Changes in MRI availability and referral practices may partly explain the lower observed incidence in the first period and the subsequent increase thereafter. Advances in cancer treatment may also have contributed by prolonging survival and increasing the time at risk for BM development. MRI availability and use, survival and the interval between primary cancer and BM diagnosis were not assessed. Competing mortality and less frequent neuroimaging may have contributed to a lower observed BM incidence in the oldest age groups. In these patients, MRI may be omitted when its findings are unlikely to alter clinical management.

This study reports crude incidence rates and age-standardized incidence rates of MRI-confirmed BM among North Savo residents over a 20-year period and describes the distribution of BM cases by primary tumor subtype in routine clinical practice.

## Conclusion

The incidence of MRI-confirmed BM among North Savo residents increased over the 20-year study period, with the trend persisting after age adjustment. Age-specific incidence peaked among individuals aged 70–74 years. Cancers originating from lung and breast tissue accounted for more than half of the identified BM cases. These findings provide regional population-based estimates of MRI-confirmed BM incidence.

## Data Availability

The datasets generated and analyzed for this work are not publicly available due to ethical and privacy restrictions related to patient confidentially but anonymized data may be reasonably requested from the corresponding author.
